# Supplementary material for: Adherence to COVID-19 preventive measures and associated factors in Ethiopia: A systematic review and meta-analysis
Source: PLoS One. 2022 Oct 13;17(10):e0275320. doi: 10.1371/journal.pone.0275320 (PMC9562213; doi:10.1371/journal.pone.0275320)
Supplement: S1 Table — (DOCX) [file pone.0275320.s001.docx]

S1 Table. Searching strategies and results; as of July 21, 2022, 11:00 am ([Western European Summer Time](https://24timezones.com/time-zone/west))

| **Databases** | **Searching terms** | **Filters** | **Number of studies** |
| --- | --- | --- | --- |
| Research4Life | (Adherence OR compliance OR associated factors OR determinants OR predictors) AND (COVID-19) AND (Preventive measures OR prevention measures OR mitigation) AND (Ethiopia) | 2019-2022, English, Journal article | 136 |
| PubMed/MEDLINE | ((((((adherence) OR (compliance)) OR (associated factor*)) OR (determinant*)) OR (predictor*) AND (english[Filter])) AND (((COVID-19) OR (COVID-19 prevent* measur*)) OR (COVID-19 mitigat*) AND (english[Filter]))) AND (Ethiopia AND (english[Filter])) Filters: English | 2019-2022, English | 438 |
| CINAHL |  |  |  |
| S1 | adherence OR compliance OR associated factors OR determinants OR predictors |  | 436,097 |
| S2 | Ethiopia |  | 7,603 |
| S3 | covid-19 prevention OR covid-19 prevention measure OR COVID-19 preventive measures OR covid-19 mitigation |  | 8,996 |
| S4 | S1 AND S2 AND S3 | English  2019-2022 | 14 |
| Web of Science |  |  |  |
| S1 | **((((ALL=(adherence )) OR ALL=(compliance )) OR ALL=(associated factors )) OR ALL=(determinants )) OR ALL=(predictors )** |  | [2,795,124](https://www.webofscience.com/wos/woscc/summary/ee434fa8-9b2d-487c-84e3-186365f2cc33-44ea490e/relevance/1) |
| S2 | **(((ALL=(preventive measures )) OR ALL=(prevention measures )) OR ALL=(mitigation )) OR ALL=(mitigative measures )** |  | [353,315](https://www.webofscience.com/wos/woscc/summary/5d82334e-9cd7-43f9-9d41-084029c4c775-44ea5f60/relevance/1) |
| S3 | **ALL=(COVID-19)** |  | [299,971](https://www.webofscience.com/wos/woscc/summary/fd445316-ebaf-4fa8-89e9-17c7df174d28-44ea65dc/relevance/1) |
| S4 | **ALL=(Ethiopia)** |  | [64,426](https://www.webofscience.com/wos/woscc/summary/a79e22a5-b5bb-496f-b59a-fc719bba65ff-44ea69ad/relevance/1) |
| S5 | **#1 AND #2 AND #3 AND #4** | 2019-2022, articles | 103 |
| ScienceDirect | **(Adherence OR compliance OR associated factors OR determinants OR predictors) AND (COVID-19) AND (preventive measures OR prevention measures) AND (Ethiopia)** | 2019-2022, research articles | 321 |
| Articles included from Google, Google scholar, JURN and other sources |  |  | 15 |
| Total |  |  | 1029 |
| Final full text relevant to our review |  |  |  |
