# Supplementary material for: Adherence to COVID-19 preventive measures and associated factors in Ethiopia: A systematic review and meta-analysis
Source: PLoS One. 2022 Oct 13;17(10):e0275320. doi: 10.1371/journal.pone.0275320 (PMC9562213; doi:10.1371/journal.pone.0275320)
Supplement: S2 Table — (DOCX) [file pone.0275320.s002.docx]

S2 Table. JBI critical appraisal checklist for analytical cross-sectional studies

| Articles | Parameter | | | | | | | | Score out of 8 |
| --- | --- | --- | --- | --- | --- | --- | --- | --- | --- |
|  | Clear inclusion criteria | Clear study setting &subject | Valid & reliable exposure measurement | Standard objective | Identification of confounding factors | Strategies to deal with confounding factors | Valid & reliable outcomes measurement | Appropriate statistical analysis |  |
| Abeya et al. | √ | √ | √ | √ | √ | √ | √ | √ | 8 |
| Asnakew et al. | X | N/A | √ | √ | N/A | N/A | √ | √ | 4 |
| Azene et al. | √ | √ | √ | √ | √ | √ | √ | √ | 8 |
| Bante et al | √ | √ | √ | √ | √ | √ | √ | √ | 8 |
| Etafa et al. | √ | √ | N/A | √ | N/A | N/A | √ | √ | 5 |
| Hailu et al. | √ | √ | X | √ | √ | √ | √ | √ | 7 |
| Kayrite et al. | √ | √ | √ | √ | √ | √ |  | √ | 8 |
| Kebede et al. | √ | √ | √ | √ | √ | √ | √ | √ | 8 |
| Keleb et al. | √ | √ | √ | √ | √ | √ | √ | √ | 8 |
| Shewasinad et al. | √ | √ | √ | √ | √ | √ | √ | √ | 8 |
| Silesh et al. | √ | √ | √ | √ | √ | √ | √ | √ | 8 |
| Temesgan et al. | √ | √ | √ | √ | √ | √ | √ | √ | 8 |
| Temesgen et al. | √ | √ | √ | √ | √ | √ | √ | √ | 8 |
| Zenbaba et al. | √ | √ | √ | √ | √ | √ | √ | √ | 8 |
| Zewude et al. | √ | √ | √ | √ | √ | √ | √ | √ | 8 |

**Key**

- X=NO, √=YES, N/A= NOT APPLICABLE,
- High methodological quality =≥5
- Low methodological quality= <5
