# Supplementary material for: Adherence to COVID-19 preventive measures and associated factors in Ethiopia: A systematic review and meta-analysis
Source: PLoS One. 2022 Oct 13;17(10):e0275320. doi: 10.1371/journal.pone.0275320 (PMC9562213; doi:10.1371/journal.pone.0275320)
Supplement: S1 Appendix — (DOCX) [file pone.0275320.s004.docx]

| **Section and Topic** | **Item #** | **Checklist item** | **Location where item is reported (Page #)** |
| --- | --- | --- | --- |
| **TITLE** | | | 1 |
| Title | 1 | Adherence to COVID-19 preventive measures and associated factors in Ethiopia: A systematic review and meta-analysis |  |
| **ABSTRACT** | | | 1-2 |
| Abstract | 2 | **Background**  Reluctance to the COVID-19 preventive measures have been repeatedly reported in Ethiopia although compliance with these actions is the key step to minimize the pandemic's burden. Hence, this systematic review and meta-analysis aims to address the gap in the literature by determining the pooled magnitude of adherence to COVID-19 preventive measures and identifying its associated factors in Ethiopia.  **Materials and methods**  The electronic databases used to search articles were PubMed/MEDLINE, CINAHL, Web of Science, ScienceDirect, Research4Life and other sources of grey literature including Google Scholar and World Health Organization (WHO) database portals for low- and middle-income countries. Full English-language articles published between 2019 and 2022 were eligible for the review and meta-analysis. Relevant data extracted and descriptive summaries of the studies presented in tabular form. The methodological quality of articles assessed using the Joanna Briggs Institute (JBI) quality assessment tool. The pooled magnitude of adherence determined by applying a random-effects model at a 95% CI.  **Results**  Of 1029 records identified, 15 articles were included in the systematic review and 11 were selected for meta-analysis. The pooled estimate of adherence to COVID-19 preventive measures in Ethiopia was 41.15% (95% CI:32.16-50.14%). Furthermore, perceived COVID-19 disease severity (AOR:1.77, 95% CI: (1.40-2.25)), attitude (AOR:1.85, 95% CI: (1.36-2.53)) and knowledge (AOR:2.51, 95% CI: (1.67-3.78)) to COVID-19 preventive measures showed significant association with adherence to COVID-19 preventive measures.  **Conclusion**  The magnitude of adherence to COVID-19 preventive measures in Ethiopia appeared to be low. Therefore, the government of Ethiopia and other stakeholders should mobilize resources to improve the adherence level of the community to the COVID-19 preventive measures and decrease public fatigue. |  |
| **INTRODUCTION** | | | 2-4 |
| Rationale | 3 | Non-adherence to COVID-19 prevention measures could have a negative impact on the health and socio-economy of Ethiopia. However, the evidence bases remained inconsistent and inconclusive. Furtherly, there is no pooled evidence concerning the adherence level to COVID-19 prevention measures and its associated factors in Ethiopia. More importantly, the systematic review and meta-analysis results will help decision-makers to plan and implement effective action against the COVID-19 pandemic. | 4 |
| Objectives | 4 | Thus, this meta-analysis aims to estimate the magnitude of adherence to COVID-19 prevention measures and its associated factors. | 4 |
| **METHODS** | | | 4-7 |
| Eligibility criteria | 5 | All English-language, full-text, original research articles conducted in Ethiopia from 2019 to 2021 and published in peer-reviewed journals or filed as completed dissertations were considered for inclusion. Moreover, the article should measure the adherence level and/or associated factors of COVID-19 prevention measures in Ethiopia. However, case series, opinion papers and reports were excluded from this meta-analysis. | 5 |
| Information sources | 6 | The electronic databases used to search articles were PubMed/MEDLINE, ScienceDirect, Web of Science, CINAHL, Research4Life and other World Health Organization (WHO) database portals for low- and middle-income countries. In addition, the researchers found related articles through a desk review of the doctoral dissertations available at Ethiopian university libraries and institutional repositories, and from reviewing the reference lists of related articles. | 4 |
| Search strategy | 7 | Electronic database searches were conducted from July 20, 2022, to July 23, 2022. The main terms used during electronic database search were: (“Adherence” OR “Compliance” OR “Associated factors” OR “Determinants” OR “Predictors”) AND (“COVID-19 prevention measures” OR “COVID-19 preventive measures”) AND (Ethiopia). | 5 |
| Selection process | 8 | All electronic search results were transferred into Mendeley reference manager software version 1. 19.8. Next, we organized all these articles into a single folder for duplicate citation removal and further ease the management of articles. After removing duplicate citations with the software, two authors (GG, RP) independently screened the articles, based on preset eligibility criteria. The article screening process had three sequential stages, title, abstract and full-text screening. Through title screening, studies entitled with terms directly/indirectly measure the adherence level and/or associated factors of COVID-19 prevention measures in Ethiopia, were selected for abstract screening. And, in abstract screening, articles were read their abstract if they could measure either of the review and meta-analysis outcomes. Consequently, full-text screenings were carried out with four independent authors. The final decision whether to include an article were reached on the consensus of all the authors. The screening and selection of articles were guided according to the PRISMA guideline (Fig 1). | 5-6 |
| Data collection process | 9 | Data extraction was carried out by two authors (GD and RP) and double checked by the other authors. | 6 |
| Data items | 10 | Data on author(s), study year, region of study, study design, sample size and factors | 6 |
| Study risk of bias assessment | 11 | Quality of studies was critically evaluated for the validity of results. The methodological quality of the papers was assessed using the JBI quality assessment checklists for cross-sectional analytical studies. This JBI critical appraisal checklist has eight elements, which mainly addresses the methodological area of each article. It focused on the appropriateness of the statistical analysis, objective, inclusion criteria, study population and setting, exposure and outcomes measurement, and management of confounding factors. The evaluation and decision of each article was based on the consensus of all authors. Accordingly, articles with positive answers (yes) for more than 50% of the eight-elemental checklist (i.e., yes for five or more) were included in this systematic review and meta-analysis | 6 |
| Effect measures | 12 | Adherence to COVID-19 prevention and associated factors (OR) | 6 |
| Synthesis methods | 13 | The raw data in the Microsoft Excel spreadsheet template was transferred to STATA^TM^ version 16 software for analysis. A pooled magnitude of adherence to COVID-19 prevention measures in Ethiopia was estimated at a 95% confidence interval (CI). Furtherly, we conducted a regional subgroup analysis. Also, a pooled odds ratio of different variables was calculated using a RevMan version 5.4.1 to check if there was an association between independent variables and the dependent variable (adherence to COVID-19 prevention measures). The heterogeneity of study outcomes was assessed using the I^2^ statistic. Accordingly, studies with high heterogeneity were estimated using a random-effects model and fixed effect model run with low heterogeneity (<50%). Parallelly, a publication bias was checked using a funnel plot asymmetry and Egger’s and Begg-Mazumdar Rank correlation tests. Eventually, the statistical analysis and the results were double-checked by all authors. | 7 |
| Reporting bias assessment | 14 | Begg’s, Egger’s tests, and trim-and-fill analysis was considered | 7 |
| Certainty assessment | 15 | Subgroup analyses by region | 7 |
| **RESULTS** | | | 8-18 |
| Study selection | 16 | A total of 1029 citations were collected through electronic database search and other sources (Fig 1). Of these, we excluded 359 items due to duplication. From the remained 670 collections, 578 items were excluded through title screening, while 70 were excluded after the abstract screening. Next, 22 full articles were reviewed according to the predefined eligibility criteria. Eventually, 15 articles were found fully eligible for systematic review, of which 11 articles were included in meta-analysis. All of them were conducted using a cross-sectional study design (10,11,13,19–30). Nearly half (7) of these studies were conducted in Amhara region (10,21,24–27,29), four articles in SNNP (Southern Nations, Nationalities, and Peoples') region (11,19,20,30), three in Oromia region (13,22,23) and the remained one study was conducted in Addis Ababa (28). The maximum sample size recorded was 2751 subjects (13), while the minimum was 324 (19). Furthermore, majority of the studies had more than 95% response rate (Table 1). | 8 |
| Study characteristics | 17 | Data on author(s), study year, region of study, study design, sample size and factors (Table 1) | 9-14 |
| Risk of bias in studies | 18 | Present assessments of risk of bias for each included study. | 15 |
| Results of individual studies | 19 | For all outcomes, present, for each study: (a) summary statistics for each group (where appropriate) and (b) an effect estimate and its precision (e.g. confidence/credible interval), ideally using structured tables or plots. | 9-15 |
| Results of syntheses | 20 | The pooled estimate of adherence to COVID-19 preventive measures in Ethiopia was 41.15% (95% CI:32.16-50.14%). Furthermore, perceived COVID-19 disease severity (AOR:1.77, 95% CI: (1.40-2.25)), attitude (AOR:1.85, 95% CI: (1.36-2.53)) and knowledge (AOR:2.51, 95% CI: (1.67-3.78)) to COVID-19 preventive measures showed significant association with adherence to COVID-19 preventive measures.  Publication bias was evidenced on both the funnel plots of precision asymmetry and the Egger’s test of the intercept. We run a trim and fill analysis in the random-effects model. The magnitude estimates did not differ significantly between the initial and, trim and fill models. | 15-18 |
| Reporting biases | 21 | Present assessments of risk of bias due to missing results (arising from reporting biases) for each synthesis assessed. | 15, |
| Certainty of evidence | 22 | Present assessments of certainty (or confidence) in the body of evidence for each outcome assessed. | 9-18 |
| **DISCUSSION** | | | 18-20 |
| Discussion | 23 | This systemic review and meta-analysis attempted to estimate the pooled magnitude of adherence to COVID-19 preventive measures and its associated factors in Ethiopia. We found 41.15% of the people were adhered to the COVID-19 preventive measures in Ethiopia. This is consistent with a previous review in the country where the pooled level of health professional’ practice, towards WHO COVID-19 management and personal protection recommendations, estimated to be 40.3%. This low level of adherence to COVID-19 preventive measures could be related with public fatigue, ignorance, misinformation, personal or social norms and perceived behavior control.  Furthermore, sex, age, perceived COVID-19 severity, attitude and knowledge to COVID-19 prevention measures were found to have a statistically significant association with adherence to COVID-19 prevention measures. Therefore, the government of Ethiopia and other stakeholders should mobilize resources to improve the adherence level of the community to the COVID-19 preventive measures and decrease public fatigue. Though, our systematic review and meta-analysis had some limitations. First, we found no qualitative studies in our review that would have explored the determinants of adherence to COVID-19 prevention measures. Secondly, even though studies are included from different regions of Ethiopia, the representativeness of the population is not as strong because all of them were observational and heterogeneous |  |
| **OTHER INFORMATION** | | | 21 |
| Registration and protocol | 24 | Not registered |  |
| Support | 25 | No funding | 21 |
| Competing interests | 26 | All authors declare that they have no conflict of interest. | 21 |
| Availability of data, code and other materials | 27 | All data generated or analyzed during this study are included as  supplementary information files. | 21 |

*From:*  Page MJ, McKenzie JE, Bossuyt PM, Boutron I, Hoffmann TC, Mulrow CD, et al. The PRISMA 2020 statement: an updated guideline for reporting systematic reviews. BMJ 2021;372:n71. doi: 10.1136/bmj.n71

For more information, visit: <http://www.prisma-statement.org/>
